# Weight Stigma and Social Media: Evidence and Public Health Solutions

**DOI:** 10.3389/fnut.2021.739056

**Published:** 2021-11-12

**Authors:** Olivia Clark, Matthew M. Lee, Muksha Luxmi Jingree, Erin O'Dwyer, Yiyang Yue, Abrania Marrero, Martha Tamez, Shilpa N. Bhupathiraju, Josiemer Mattei

**Affiliations:** ^1^Department of Nutrition, Harvard T.H. Chan School of Public Health, Boston, MA, United States; ^2^Brigham and Women's Hospital, Boston, MA, United States

**Keywords:** weight stigma, social media, disordered eating, obesity, weight bias internalization

## Abstract

Weight stigma is a pressing issue that affects individuals across the weight distribution. The role of social media in both alleviating and exacerbating weight bias has received growing attention. On one hand, biased algorithms on social media platforms may filter out posts from individuals in stigmatized groups and concentrate exposure to content that perpetuates problematic norms about weight. Individuals may also be more likely to engage in attacks due to increased anonymity and lack of substantive consequences online. The critical influence of social media in shaping beliefs may also lead to the internalization of weight stigma. However, social media could also be used as a positive agent of change. Movements such as Body Positivity, the Fatosphere, and Health at Every Size have helped counter negative stereotypes and provide more inclusive spaces. To support these efforts, governments should continue to explore legislative solutions to enact anti-weight discrimination policies, and platforms should invest in diverse content moderation teams with dedicated weight bias training while interrogating bias in existing algorithms. Public health practitioners and clinicians should leverage social media as a tool in weight management interventions and increase awareness of stigmatizing online content among their patients. Finally, researchers must explore how experiences of stigma differ across in-person and virtual settings and critically evaluate existing research methodologies and terminology. Addressing weight stigma on social media will take a concerted effort across an expansive set of stakeholders, but the benefits to population health are consequential and well-worth our collective attention.

## Introduction

The prevalence of weight discrimination has increased dramatically in the United States (US), as much as 66% between 1995 and 2006 (1), and 71% of adolescents reported being bullied about their weight in the past year (2). Weight stigma, also commonly referred to as “weight bias,” “weight discrimination,” or “weight prejudice” (3), refers to the labeling, stereotyping, separation, and discrimination of individuals, populations, and organizations on the basis of weight (2). While conversations about weight stigma have historically centered on individuals who are classified with overweight or obesity, evidence suggests that those classified as underweight also experience stigma that exacerbates poor health (4–7). Experiences of weight stigma manifest across different contexts, but there is growing recognition of the role of social media in both promoting and reducing weight stigma, especially amongst youth. The use of social media, which refers to internet-based platforms that enable people to connect virtually to share experiences, information and ideas (8), has become ubiquitous. More than 3.6 billion people are connected worldwide, and this number is projected to increase to 4.41 billion by 2025 (9). Problematic social media use has become especially concerning during the COVID-19 pandemic (10–14). Emerging research on “#quarantine15,” a hashtag on platforms including Instagram and Twitter that features stigmatizing-posts related to weight gain during the pandemic, shows that individuals may be increasingly exposed to content that emphasizes thinness as a normative ideal and perpetuates disputed notions of the self-controllability of weight (10, 14). Given the need for greater clarity and social media saturation, this review aimed to synthesize the evidence surrounding the intersections of weight stigma and social media and discuss implications for policy, industry, practitioners, and research.

## Weight Stigma and Health

Understanding the mechanisms by which weight stigma inhibits health and well-being is critical to any discussion of the relationship between weight stigma and social media. These pathways are cyclical in nature, and as outlined by Tomiyama, involve the influence of weight stigma on stress, disordered eating, homeostatic imbalance, and subsequent weight change (15). First, weight stigma correlates with adverse indicators of physical health. For example, a randomized trial of US women conducted by Major et al. found that weight stigmatizing scenarios led to reduced executive functioning (16). Weight stigma has been associated with higher likelihood of developing key risk factors for chronic disease. Randomized studies of women by Himmelstein et al. and Schvey et al. found that exposure to stigmatizing statements or conditions resulted in elevated levels of cortisol (17, 18), a glucocorticoid that inhibits inflammation clearance (19) and is associated with the development of hypertension, hyperglycemia, insulin resistance, and hyperlipidemia (20). Other studies, including a 10-year longitudinal analysis of U.S. adults in the MIDUS Biomarker Substudy by Vadiveloo and Mattei, suggests that perceived weight discrimination may be associated with chronic health conditions such as allostatic load, glucose dysregulation, inflammation, atherosclerosis, diabetes, hypercholesterolemia, myocardial infarctions, and stomach ulcers (21–26). However, the causal relationships between weight stigma and distal health outcomes remain unresolved.

Weight stigma may also drive adverse psychosocial health outcomes, such as anxiety, depression, body image dissatisfaction, and negative self-esteem (20). Using a structural equation mediation modeling approach with a diverse sample of US adults, Himmelstein et al. found that perceived weight stigma was related to intermediary outcomes such as exercise avoidance, maladaptive eating, lifestyle behaviors, and negative affect, which in turn were associated with depressive symptoms, poorer physical health, and higher frequency of dieting. (27). A recent meta-analysis of 22 studies across various countries by Zhang et al. found an adverse relationship between use of social networking sites and disordered eating behaviors (28). For example, individuals exposed to weight-related stigma in experimental settings have been shown to have increased *ad libitum* total energy intake (29–31), to be more likely to binge eat and less likely to adhere to weight loss treatments (32, 33), and to be less likely to engage in physical activity (31, 34–39).

These relationships seem to hold true across genders (40), but heterogeneity by race/ethnicity exists. While weight stigma is similarly prevalent across different racial/ethnic groups, Black men and women report lower weight bias internalization compared to White men and women. However, a cross-sectional analysis of US adults found that Black men and Hispanic women were more likely to cope with stigma by disordered eating relative to White men and women, suggestive of distinct internalizing mechanisms and social norms across racial/ethnic groups (41). Weight stigma also varies across the life course. For example, pregnant women classified with higher body mass index (BMI) are more likely to report stigmatizing interactions with their providers and lower satisfaction with care (42), which in turn may prevent them from seeking additional health care resources (43). In addition, the high prevalence of weight stigma among youth is well-documented, and evidence suggests that children who experience weight-based teasing or bullying are more likely to binge eat or decrease levels of physical activity, and are at higher risk of developing obesity or overweight in adolescence (2). This intersectionality complicates our conceptualization of weight stigma and highlights the need for high-quality studies that assess the effects of weight stigma in diverse populations, as well as the different ways in which social media use may mediate this relationship (44). Because social media use is becoming increasingly accessible as technologies become less expensive to produce and purchase, interventions that aim to improve population health by addressing weight stigma exposure on social media may be cost-effective and wide-reaching.

## Influence of Social Media on Weight Attitudes and Stigma

Social media, such as Instagram, Facebook, or Twitter, refers to online, virtual-based platforms that allow individuals to connect and share content, either in private or public capacities (8). Due to its widespread use and theoretical ability to connect individuals across different experiences and perspectives, social media has a potentially prominent role in both exacerbating and lessening weight stigma. The utility of social media and networks as a valuable tool in shaping health attitudes and behaviors is well-established, particularly in the case of adolescent smoking. The “truth” campaign, which uses a counter-marketing strategy to reduce initiation and improve cessation of youth smoking by counteracting tobacco advertising seen by adolescents on digital media, has organized initiatives on TikTok (45), Vine (46), Facebook (47), Instagram (48), Twitter (49), and LinkedIn (50), and has shown to be effective in increasing awareness for and shifting youth attitudes against tobacco and tobacco companies (51–56). Similarly, social media may have both negative and positive influences on weight stigma (Table 1).

**Table 1:** Summary of the positive and negative influences of social media on weight stigma and body shape perceptions.

**Negative**	**Positive**
Lead to unintended censorship of activists and educators due to user-determined reporting systems and imperfect content moderation algorithms (57, 58).	Provide opportunities for the spread of social movements aimed at increasing body positivity, self-acceptance, and advocacy (59–63).
Expose individuals to a high degree of stigmatizing posts and comments without adequate filtering or flagging of potentially problematic content (64, 65).	Raise awareness for weight bias, stigmatization, and discrimination as increasingly common in popular culture (59).
Facilitate cyberbullying due to increased anonymity, lack of real consequences, and reach (66–68).	On public channels, provide easier opportunities to support individuals targeted by cyberbullying and reduce likelihood of bystander effects (69).
Increased weight dissatisfaction, drive for thinness, and thin ideation, leading to internalization of weight stigma (65, 70–72).	Increase acceptance of diverse body sizes, self-esteem, and mood when body acceptance related advertising or content is viewed (73).
Lead to increased concerns about being judged by others, and emphasizing external determinants of self-worth (74, 75).	Provide opportunities for self-expression and identity formation (75).
Increase social isolation and anxiety due to the sheer volume of content, time demands, and perceived social obligations, and increasing opportunities for social exclusion (75).	Increase social inclusion among populations who would normally be marginalized in society, increasing feelings of belonging and well-being (75–78).
Inadvertent exposure to triggering news, events, and products that lead to negative affect (75).	Improve adherence and effectiveness of weight management interventions by increasing communication and social support, especially for those that lack in-person support systems (79–84).
Increase feelings for peer competition and augment the effect of peer competition on increased body dissatisfaction (75, 85).	Online interventions and social media campaigns may be more effective in reaching youth and adolescents who may be more technologically adept and for whom traditional intervention approaches have previously failed (70).

### Negative Influences of Social Media

Using ecological theory, weight stigma can be characterized into three distinct subtypes: structural, interpersonal, and intrapersonal (3). Negative influences of social media may manifest across each of these levels and ultimately contribute to poor health. *Structural* weight stigma describes weight discrimination and negative beliefs encoded into societal systems such as medical institutions and consumed media. On social media, structural weight bias may lead to unintended algorithmic or policy decisions that filter out or flag content from individuals in stigmatized weight classes. For example, some have speculated that an algorithm designed to censor inappropriate images on Instagram, a platform now owned by Facebook which itself has recently been publicly criticized for censorship and misinformation concerns (86), has led to posts by larger-bodied “influencers” being disproportionately targeted for removal and to the selective filtering of content shown to users of a platform that may reflect normative ideals of weight (57, 58). These algorithms might have a “silo-ing” effect for consumers that leads to repeated exposure to stigmatizing content and reinforces harmful norms about weight (70). Because these algorithms are proprietary and their mechanisms opaque, research on structural forms of weight stigma on social media is largely absent.

*Interpersonal* experiences of stigma derive from person-to-person interactions. This may be especially problematic in virtual settings where a higher degree of anonymity can reduce the influence of normative beliefs and subjective norms in an individuals' decision to engage in cyberbullying (66–68). For example, Jeon et al. found that attacks on individuals classified as overweight were twice as frequent as comments in their defense on YouTube (64). A content analysis by Chou et al. of 1.37 million posts from major social media sites and their respective comments found that 92% of posts related to obesity used the term “fat,” and that these posts were more often associated with negative, derogatory, and misogynist connotations (65). In addition, the authors found that the large majority (91.5%) of all obesity-related interactions occurred on Twitter (65). These negative weight-based aggressive comments have feedback effects (15), resulting in greater risk of depression and anxiety in individuals classified as overweight. For example, a cross-sectional analysis of adults with obesity in the US by Friedman et al. found that higher frequency of interpersonal stigmatizing experiences was correlated with more severe phobic anxiety as assessed by the Symptoms Checklist-90-R (SCL-90-R) and higher Beck Depression Inventory scores (87).

Last, *intrapersonal* weight stigma refers to the internalization and embodiment of weight bias into an individual's beliefs and perceptions about their abilities and intrinsic worth. Social media plays a key role in social, cultural, and political identity construction (88, 89), such that individuals may develop weight-related attitudes and beliefs that reflect their differential exposure to stigmatizing or non-stigmatizing online content. For example, social media may spread information that focuses on behavioral or lifestyle determinants (e.g., diet or physical activity) of weight change, rather than on more upstream environmental or social factors such as obesogenic neighborhood retail or food environments. In addition, social media may serve to augment existing norms on thinness and body image that are driven by peer comparison and competition. In an analysis of 237 pre-adolescent and adolescent girls, Ferguson et al. found that social media use at baseline was positively correlated with greater feelings of peer competition, which were in turn positively correlated with body dissatisfaction and eating disorder symptoms at follow up (85). In a content analysis of nearly a thousand tweets following a major fashion show, Chrisler et al. found that users of social media platforms frequently engaged in upwards social comparisons to models and expressed sentiments related to body dissatisfaction, disordered eating, and self-harm (90). Social comparison may also occur among peers on social media. For example, Fardouly et al. found that the frequency of comparison to peers on Facebook mediated the positive association between Facebook usage and body image concerns (91).

Food industry and lifestyle groups are also more likely to promote purchasable items on Facebook and Instagram, and health promotion organizations are more likely to feature information about fruits, vegetables, and grains (92). While each entity may have different motives, the key assumption behind the types of messages users may see on social media is that the locus of responsibility is centrally on the individual, rather than on corporations, governments, or society. As a result, people living in large bodies may be implicitly or explicitly portrayed in stigmatizing ways as unintelligent or undisciplined (65, 71). Messaging on social media may promote individual blame beliefs, leading to negative self-perception and internalization of stigma in individuals with underweight, overweight, or obesity classifications and increase the likelihood of maladaptive coping behaviors that may persist over time (73). Exposure to “thin-ideal” images and advertising are also prevalent across different forms of media. In a randomized study of 475 female students over 18 years of age in the United States, Selensky and Carels found that exposure to thin-idealizing advertising resulted in greater dislike of persons with overweight and obesity and lower self-satisfaction of body shape or weight (73). In another randomized study of college-age women enrolled in classes at Utah State University by Hawkins et al., exposure to thin-ideal magazines elicited higher body dissatisfaction, negative affect, eating disorder symptoms, and decreased self-esteem (72).

### Positive Influences of Social Media

While social media may exacerbate experiences of stigma, it may also serve to provide a space to build solidarity, reduce social isolation, and increase awareness of weight bias. The Body Positivity (BoPo) movement is a prominent social movement to reduce weight bias on social media, which, while largely decentralized, has been heralded by some influential personas (59). BoPo, which has roots in the 1960s feminist fat-acceptance movements in the US, aims to challenge and subvert traditional ideals about beauty by promoting body appreciation, acceptance, and self-empowerment (60–63). Unsurprisingly, the BoPo movement has predominantly focused on social media sites that feature images. Viewing BoPo content is associated with lower negative affect and higher positive affect and body satisfaction among college students, while exposure to “thinspiration” content that emphasizes thin-ideals is associated with lower body satisfaction, higher negative affect, and lower positive affect (60). In experimental settings, randomized exposure to body positive photos, quotes, and no-makeup selfies has been shown to result in better mood and self-perception with larger benefits seen among participants with disordered eating symptoms or low self-compassion (93, 94). For example, an experimental study conducted by Cohen et al. found that women who were randomized to view body-positive Instagram posts were more likely to express positive mood, body satisfaction, and body appreciation, relative to those who were randomized to view thin-idealizing or neutral content (95). However, some have raised concerns on whether the BoPo movement may inadvertently perpetuate evaluations of self-worth that are dependent on appearance, pointing to the commodification and co-opting of the movement by large corporations that use the platform to promote the sale of their products (74, 96–99), and have instead proposed a “radical body positive” that seeks to critique how we are expected to understand and inhabit our bodies in the first place (74).

A second typology of movements on social media that aims to address weight stigma involves reclamation of the term “fat” in order to challenge traditional connotations of the word. For example, “Notes from the Fatosphere (i.e., Fatosphere),” an online community where individuals who self-identify as “fat” share their experiences and promote health without a focus on weight loss, has shown to foster a sense of inclusion, empowerment, and well-being, and to help individuals better cope with stigmatizing situations (76, 77). The “Health at Every Size (HAES)” movement, present on Instagram and TikTok, also encourages a weight-neutral approach to health and aims to promote self-acceptance across a diversity of body sizes, though content analyses suggest that posts using the hashtag may still contain stigmatizing sentiments (100). Clearly, social media has the potential to positively impact behaviors and attitudes, and researchers will need to continue to evaluate the influence of structure and content in real-world applied settings to assess the most effective type of messaging. These efforts may be simultaneously supported by policies and programs implemented by social media platforms and governments that reduce the accessibility of stigmatizing content.

## Implications for Policy and Industry Practice

### Public Policies and Programs

Because of the demonstrated detrimental effects of weight stigma on individuals' nutritional habits and physical and mental well-being, social media public policy development should be a central concern and advocacy focus for companies, public health professionals, parents, educators, and policymakers. The vast reach of social media globally and the prevalence of use among teens and young adults (101) suggest that problematic social media use is both a concern and a potential area for intervention. However, only a few states, such as Massachusetts (102), have proposed legislation that would codify weight-based discrimination as illegal. Michigan is the only example where such policy has been passed into law (103). While potential laws prohibiting weight discrimination in the US have widespread public support (104–106), it remains unknown how such a policy would regulate social media platforms and their users. Given apparent political difficulties in successfully legislating to regulate weight-based discrimination, industry self-regulation and accountability can be key intermediate alternatives to protect vulnerable populations. Potential options for industry self-regulation are discussed below.

### Industry Practices

The majority of comments directed toward individuals living in larger bodies on social media express negative sentiment (107), which can drown out positive health messages from public health officials and institutions as well as undermine individuals who seek social support via social media in their efforts to lose weight and/or adopt healthier lifestyles. Big Tech has come under criticism recently for its failure to limit the spread of misinformation (108), hate speech (109), and racial bias (110), as well as for its active role in suppressing content from users deemed physically unattractive, lower income, or disabled in the case of TikTok (111). Anti-weight bias advocates can capitalize on this momentum to reform how companies operate. First, weight bias and discrimination must be included in the articulated community guidelines outlined by social media platforms. While most platforms have policies that flag or prohibit posts that include potentially harmful or offensive content (Table 2) such as violence, suicide, bullying, eating disorders, misinformation, or hate speech (112, 116, 123), enforcement is especially absent on newer platforms (124). Recently, however, Pinterest has proposed policies that limits forms of bias, shame or discrimination based on individuals' body size or weight in content, as well as those that prohibit the advertisement of weight loss products, their testimonials, and the use of language idealizing certain body types (121, 122, 125). Meanwhile, Facebook's policy on hate speech covers content related to race, gender, sexual orientation, religious affiliation, and disease/disability, not weight stigma or body image bias (112). Pinterest's new policies ought to act as a model for change, motivating other social media companies to re-invest in developing robust content moderation teams that feature diverse experiences and backgrounds with adequate training in identifying weight stigmatizing content. Similar training is already available for medical professionals (126, 127), and even modest interventions have been shown to improve beliefs about individuals classified with obesity or overweight (128). Last, training data for machine learning algorithms that are used to identify potentially discriminatory content should include labels for weight stigmatizing material. For example, the algorithm could help prevent offensive comments and posts before they are posted by showing users prompts suggesting their comment might be harmful to others and asking if they would like to rephrase (112, 129, 130). Recent analyses of innovative hate speech detection algorithms have shown promise in identifying and reducing racial bias, and similar strategies could be applied for weight (131). Together, these technologies could be leveraged to prevent weight stigma by identifying words and word combinations (e.g., “fat” and “lazy” in the same comment) associated with fat-shaming and weight stigmatization through a process of algorithmic detection, human review, and subsequent removal.

**Table 2:** Examples of relevant existing content moderation policies on social media (June 17, 2021).

**Platform**	**Policy**	**Scope**
Facebook	Hate speech (112)	Content that includes hate speech against protected characteristics (race, ethnicity, national origin, disability, religious affiliation, caste, sexual orientation, sex, gender identity, serious disease) are subject to review and removal.
	Violent and graphic content (113)	Content that glorifies violence or celebrates the suffering or humiliation of others subject to remove. Warning labels added to especially graphic or violent content so that users under age 18 cannot view.
	Adult nudity and sexual activity (114)	Content that includes real nude adults, sexual activity, or fetish content is subject to removal, though allowances for content shared as a form of protest, to raise awareness, or for educational purposes may be allowed depending on reviewed intent.
Instagram	Hate speech, bullying, and abuse (115)	Credible threats of violence, hate speech, or targeting of private individuals on the basis of race, ethnicity, national origin, sex, gender, gender identity, sexual orientation, religion, disability, or disease subject to removal.
	Appropriate imagery (115)	Nudity, with some exceptions (post-mastectomy scarring, breastfeeding, paintings, sculptures) subject to removal.
	Self-injury (115)	Glorification or encouragement of self-injury, including eating disorders, is not allowed.
Twitter	Hateful conduct policy (116)	Promotion of violence targeted against people on the basis of race, ethnicity, national origin, cast, sexual orientation, gender, gender identity, religious affiliation, age, disability, or serious disease subject to review, including hateful imagery.
	Sensitive media policy (117)	Graphic violence, adult content (full or partial nudity, simulated sexual acts, sexual intercourse), violent sexual conduct, gratuitous gore, and hateful imagery subject to warning, removal, and/or account suspension.
TikTok	Hateful behavior (118)	Attacks on the basis of protected attributes (race, ethnicity, national origin, religion, caste, sexual orientation, sex, gender, gender identity, serious disease, disability, immigration status), slurs, and hateful ideology subject to removal and account ban.
	Suicide, self-harm, dangerous acts (118)	Imagery that depicts self-harm or eating disorders subject to removal, regardless of intent.
	Harassment and bullying (118)	Content with abusive behavior (threats, degrading statements), unwanted or inappropriate sexual behavior directed at another individual, and threats to hack, dox, or blackmail, subject to removal
	Adult nudity and sexual activities (118)	Nudity and sexual activity, including depictions of nudity or sexual acts, may be removed pending review procedure.
Reddit	Promoting hate based on identity or vulnerability (119)	Users and communities that incite violence or that promote hate based on identity or vulnerability may be banned, including groups based on their perceived race, color, religion, national origin, ethnicity, immigration status, gender, gender identity, sexual orientation, pregnancy, or disability.
	Do not threaten, harass, or bully (120)	Harassment, threatening, or bullying of people by individuals or communities not tolerated, though dependent on self-report. Content moderation largely handled by user-nominated “community mods”
Pinterest	Hateful activities (121)	Limits the distribution of or removes hateful content against protected or vulnerable groups, defined as people grouped together on the bases of perceived race, color, caste, ethnicity, immigration status, national origin, religion or faith, sex or gender identity, sexual orientation, disability or medical condition, socio-economic status, age, weight or size, pregnancy, or veteran status.
	Adult content (121)	Limits the distribution of or removes mature and explicit content, including fetish imagery, vivid sexual descriptions, graphic depictions of sexual activity, and images of nudity where the poses, camera angles, or props suggest pornographic intent.
	Harassment and criticism (121)	Limits the distribution of or removes insulting content including manipulated imagery intended to degrade or shame, shaming people for their bodies or assumed sexual or romantic history, sexual remarks about people's bodies, criticisms involving name-calling or profanity, or mocking someone for experiencing sadness, grief, loss, or outrage.
	Self-injury and harmful behavior (121)	Limits the distribution of or removes content that displays, rationalizes, or encourages suicide, self-injury, eating disorders or substance abuse.
	Weight loss products and services (122)	Prohibits weight loss ads and ads that body shame, including weight loss language or imagery, testimonials regarding weight loss or weight loss products, any language or imagery that idealizes or denigrates certain body types, referencing BMI or similar indices, weight loss or appetite suppressant pills, any products that claim weight loss through something worn or applied to skin, before-and-after weight loss imagery, weight loss procedures like liposuction or fat burning, body shaming such as imagery or language that mocks or discredits certain body types or appearances, and claims regarding unrealistic cosmetic results.

## Implications for Practitioners and Researchers

### Implications for Public Health Practitioners and Clinicians

Public health practitioners and clinicians must grapple with the tension between promoting body positivity while helping patients understand that behaviors associated with an unhealthy body mass are strong risk factors for morbidity and mortality. This is especially crucial given the pervasiveness of weight bias among medical students (132) and medical professionals, even those who specialize in treating individuals with obesity (133). Therefore, practitioners and clinicians have a pivotal role in encouraging healthy (both physical and psychological) weight maintenance. Some evidence suggests that leveraging the social network basis of many social media sites including Facebook or Instagram can augment and support healthy weight interventions. For example, Facebook based peer-group interventions have been shown to be effective in improving feeding practices among families with infants at high risk for developing obesity (79), and in improving parent engagement in a preschool obesity prevention curriculum among households participating in Head Start (80). Clinicians or counselors, who may actively monitor or even lead these interactions on social media (81), should receive training in weight bias and ensure that instances of weight stigmatization are absent. As a general practice, clinicians should aim to increase patient awareness of the pervasiveness of weight stigma on social media so that individuals can identify problematic ideals and content themselves. Mental health clinicians should also receive the appropriate resources and training to recognize sources of weight stigmatization, both in their own use of language as well as in their patients' experiences, that may contribute to mental health diagnoses, especially given the role of social media in identity formation.

### Implications for Research

Measurement of individuals' exposure to stigmatizing situations has typically ignored differences by online vs. in-person settings. For example, the commonly used Myers and Rosen Stigmatizing Situations Inventory includes assessment of weight-related stigma in public spaces, in medical settings, at school, at work, and at home (134, 135). Because digital interactions are increasingly common (136), and because online social interactions may be distinctly different than in-person social interactions due to increased anonymity, non-verbal cues, and the ability to easily connect across physical geographies (137–139), research that relies on participant questionnaire data should consider developing and implementing measures that assess exposure to weight stigma on social media specifically.

A recent joint international consensus statement on ending weight stigma, published by a panel of 36 experts across diverse disciplines and institutions, emphasized the need for academic institutions, professional organizations, the media, public health authorities, and governments to receive education on weight stigma consistent with current scientific knowledge (140). A natural extension, and one that has received little attention in the literature, is an evaluation of the ways in which research methods themselves might expose participants to stigmatizing scenarios. A survey of forty questionnaires and scales used in weight stigma research by Lacroix et al. found that only one used people-first language, and measures evaluating weight bias internalization frequently included terms such as “my weight,” “my weight problems” or “being overweight” (3). In solidarity with critiques of racism, ableism, sexism, and genderism in research (141–145), investigators should evaluate how weight terminology in their work and methods implicitly stigmatizes participants and alters responses. Doing so acknowledges the presence of weight stigma in other facets of life, including on social media, while attempting to minimize any additional burden to populations participating in scientific studies.

## Conclusion

Weight stigma describes an unfounded societal value placed on individuals based on appearance and anthropometry. It can be detrimental to one's physical and psychological health. Social media serves as both a platform fostering weight stigma and body shaming, as well as a safe haven for communities to find support and body positivity promotion. While policies are in place to mediate harmful dialogue on these various platforms, there remains opportunity for improved sensitivity within nutrition communications and within the medical field alike. The health impact of imposed weight stigma on social media is of increasing public health concern and warrants further research and action.

## Author Contributions

OC and ML conceptualized the topic, researched and analyzed the background literature, and wrote the manuscript, including interpretations. MLJ, EO'D, and YY researched and analyzed the background literature and wrote portions of the manuscript, including interpretations. AM, MT, SB, and JM provided substantial scholarly guidance on the conception of the topic, manuscript draft and interpretation, and revised the manuscript critically for intellectual content. All authors contributed to the article and approved the submitted version.

## Conflict of Interest

The authors declare that the research was conducted in the absence of any commercial or financial relationships that could be construed as a potential conflict of interest.

## Publisher's Note

All claims expressed in this article are solely those of the authors and do not necessarily represent those of their affiliated organizations, or those of the publisher, the editors and the reviewers. Any product that may be evaluated in this article, or claim that may be made by its manufacturer, is not guaranteed or endorsed by the publisher.
